# Bacterial Phytochromes, Cyanobacteriochromes and Allophycocyanins as a Source of Near-Infrared Fluorescent Probes

**DOI:** 10.3390/ijms18081691

**Published:** 2017-08-03

**Authors:** Olena S. Oliinyk, Konstantin G. Chernov, Vladislav V. Verkhusha

**Affiliations:** 1 Department of Biochemistry and Developmental Biology, Faculty of Medicine, University of Helsinki, 00290 Helsinki, Finland; olena.oliinyk@helsinki.fi (O.S.O.); konstantin.chernov@helsinki.fi (K.G.C.); 2 Department of Anatomy and Structural Biology, and Gruss-Lipper Biophotonics Center, Albert Einstein College of Medicine, Bronx, NY 10461, USA

**Keywords:** bacterial photoreceptor, near-infrared fluorescent protein, phytochrome, cyanobacteriochrome, allophycocyanin, tetrapyrrole

## Abstract

Bacterial photoreceptors absorb light energy and transform it into intracellular signals that regulate metabolism. Bacterial phytochrome photoreceptors (BphPs), some cyanobacteriochromes (CBCRs) and allophycocyanins (APCs) possess the near-infrared (NIR) absorbance spectra that make them promising molecular templates to design NIR fluorescent proteins (FPs) and biosensors for studies in mammalian cells and whole animals. Here, we review structures, photochemical properties and molecular functions of several families of bacterial photoreceptors. We next analyze molecular evolution approaches to develop NIR FPs and biosensors. We then discuss phenotypes of current BphP-based NIR FPs and compare them with FPs derived from CBCRs and APCs. Lastly, we overview imaging applications of NIR FPs in live cells and in vivo. Our review provides guidelines for selection of existing NIR FPs, as well as engineering approaches to develop NIR FPs from the novel natural templates such as CBCRs.

## 1. Introduction

Discovery of genetically engineered fluorescent proteins significantly accelerated development of modern biology. The choice of a fluorescent protein (FP) for particular imaging application depends on its properties, including molecular weight, oligomeric state, availability of chromophore and tissue penetration of exciting and emitted light. Although green fluorescent protein (GFP) and GFP-like family are widely applied to diverse areas of imaging, there are several applications where their performance is limited [1]. Particularly, absorbance and fluorescence of GFP-like FPs are restricted to visible part of a spectrum that substantially affects their efficiency in in vivo applications. To overcome these limitations, various natural photoreceptors were employed as molecular templates for FP engineering. The photoreceptor-derived FPs have been successfully applied to report dynamics and interaction signaling molecules in time and space [2]. Furthermore, photoreceptors have been used to design biosensors and optogenetic tools to monitor and control various intracellular processes including gene expression, protein phosphorylation and degradation, a flux of calcium and other ions [3,4].

Noninvasive imaging techniques require minimal interference with normal cellular metabolism. To achieve this, engineered FPs should efficiently fold and incorporate a chromophore available in a cell. Flavin chromophores are readily available in different tissues and organisms due to their role as cofactors in redox reactions. Cobalamin (vitamin B_12_) is involved in the synthesis of DNA, and fatty and amino acids, and is available in a large amount in tissues. Linear tetrapyrrole biliverdin (BV), which is a product of heme degradation, is also present in mammalian tissues. Conversely, the availability of linear tetrapyrrole phycocyanobilin (PCB) is restricted to plants and cyanobacteria. Therefore, FPs engineered from photoreceptors should incorporate chromophores presented in significant quantities in target tissue [3,4]. 

Light penetration is the most critical factor that must be considered for deep tissue imaging. It is of little concern for tissue culture or single-cell organism studies but it can be a limiting factor for experiments in whole animals. Particularly, imaging deeply in brain poses a major technical challenge and requires reagents that are visible at a centimeter depth. 

Visible light is significantly scattered by lipids and absorbed by hemoglobin and melanin presented in mammalian tissues, whereas near-infrared (NIR) light within the spectral region of 650–900 nm, known as the “NIR tissue transparency window”, penetrates significantly deeper. Although some GFP-like FPs exhibit substantially red-shifted emission, their excitation light is still strongly absorbed by tissues. One way to overcome that is to use two-photon excitation. However, because the large part of the emission spectra still lays outside the NIR window their imaging depth is rather limited. In this regard, FPs engineered from bacterial phytochrome photoreceptors (BphPs) are superior because their absorption and emission lay both in NIR [4,5,6].

In addition to spectral characteristics, other crucial parameters of FPs include molecular brightness (a product of extinction coefficient and quantum yield), photostability and cellular (also referred to as effective) brightness. The latter depends on the efficiency of protein folding, intracellular stability and efficiency of incorporation of the endogenous chromophore. Importantly, other intracellular compounds can compete with chromophore for binding to FP apoform. For example, heme-derived compounds, such as protoporphyrin IX (PPIX), compete with BV for incorporation to BphP-derived FPs and significantly decrease their brightness [7]. Increased brightness is often required for the detection of sparse intracellular targets, whereas, for the long-term imaging of fusions, photostability may be of crucial importance. Therefore, particular experimental design determines the choice of an FP. 

NIR FPs are superior for multicolor imaging because they complement existing palette of GFP-like FPs. Moreover, a combination of blue light-sensing optogenetic tools and NIR FPs enables spectral multiplexing [8]. The different spectral properties of NIR FPs allow researchers to separate several populations of cells by flow cytometry using visible red and NIR lasers [9]. Multiple cell populations labeled with multi-color NIR FPs can be separated in tissue cultures and in living mice using a linear spectral unmixing [10]. Excellent spectral compatibility of GFP-like FPs and NIR FPs allowed visualization and quantification of tumors both in vivo and ex vivo, as well as the evaluation of anti-cancer treatments [11]. Therefore, multicolor imaging accelerated the establishment of the novel in vivo models for various types of diseases. Furthermore, a combination of NIR bioluminescence and NIR fluorescence enables multi-modal imaging for detecting of cancer cells at different scales, from whole organs in vivo to individual metastatic cells ex vivo [12].

Here we first describe phenotypes, photoconversion mechanisms and spectral properties of photoreceptors used as molecular templates for NIR FP engineering. We then focus on experimental approaches employed for the development of NIR FPs. We next provide an overview for multiple applications of NIR FPs in basic biology and biomedicine. Lastly, we discuss perspectives of NIR FP development and their future application in imaging.

## 2. Photoreceptors as Molecular Templates for Engineering of Fluorescent Proteins

### 2.1. Structure and Photoconversion of Bacterial Phytochromes

Phytochromes were initially discovered in plants where they act as proximity sensors to modify plant growth and development, constituting the “shade-avoidance syndrome”. Plant phytochromes are able to photoconvert between far-red-light absorbing inactive state, termed Pr (λ_max_ = 660 nm), and near-infrared-light absorbing active state, termed Pfr (λ_max_ = 730 nm) [13]. In ground Pr state, absorption maximum of plant phytochromes is close to that of chlorophylls. Upon vegetation, the intensity of far-red light significantly decreases due to its absorption by photosynthetic components. This spectral change causes accumulation of inactive Pr state of plant phytochromes. In the response, shade-avoiding plants have enhanced elongation growth to exhibit their leaves to light [14,15]. 

In addition to plants, phytochromes are found in many kingdoms of life, including cyanobacteria, algae, fungi and bacteria, but not in higher animals or archaea. In bacteria, phytochrome family members play important roles in the intracellular signaling, regulating expression of respiration and photosynthetic complexes [16]. All phytochromes were structurally classified into three subfamilies according to the number of domains in their photosensory core module (PCM). Members of the most common “canonical” phytochrome subfamily contain three domains in their PCM, such as PAS (Per-ARNT-Sim), GAF (cGMP phosphodiesterase-adenylate cyclase-FhlA) and PHY (phytochrome-specific domain). Although the amino acid sequences of these domains show low sequence similarity, their structures share a common topology. Another two subfamilies include cyanobacterial phytochromes (Cph), which lack an N-terminal PAS domain, and cyanobacteriochromes (CBCRs), which contain a single GAF domain [17]. The PAS, GAF and PHY domains interact with each other and mediate protein-protein interactions that result in the formation of homo- and heterodimers with parallel, antiparallel and butterfly-like orientation of subunits [18,19,20,21]. 

The PAS domain contains about 110 amino acid residues and adopts three-dimensional structure consisted of antiparallel β-sheet flanked by α-helices. This PAS fold is found in proteins from a wide variety of prokaryotic and eukaryotic organisms [22]. These include voltage and ion channels, protein kinases, phosphodiesterases and eukaryotic transcriptional factors regulating development, cell division and circadian rhythms. In these proteins, PAS domains act as sensor moieties connected to various output modules by α-helical linkers serving as wires for signal transduction. The PAS domain binds to various cofactors, either covalently or non-covalently, allowing receptors to respond to a diversity of chemical signals or physical stimuli such as light [23]. For example, the LOV (light, oxygen, voltage) domains, which is a subclass of the PAS domains, bind a flavin chromophores and sense blue light [24], whereas the PAS domains in BphPs covalently bind BV and play a role in perception of far-red and NIR light. The most prominent structural property of canonical phytochromes is a knot structure in which the N-terminus of the PAS domain is threaded through the elongated loop of the GAF domain.

The GAF domain of phytochromes plays the major role in light perception because it has a cavity between the β-sheet and three α-helices that binds a chromophore. PAS and GAF domains share the common fold and are closely integrated with each other by the knot structure. These domains are then connected to PHY domain by long continuous α-helices that form a helical bundle. A notable structural feature of PHY domain is evolutionally conserved hairpin structure, termed “PHY-tongue”, which is in a direct contact with the chromophore. PHY-tongue shields the chromophore from the solvent and undergoes structural rearrangement upon Pr↔Pfr photoconversion, which is further propagated through the phytochrome molecule [21,25].

**Figure 1:**
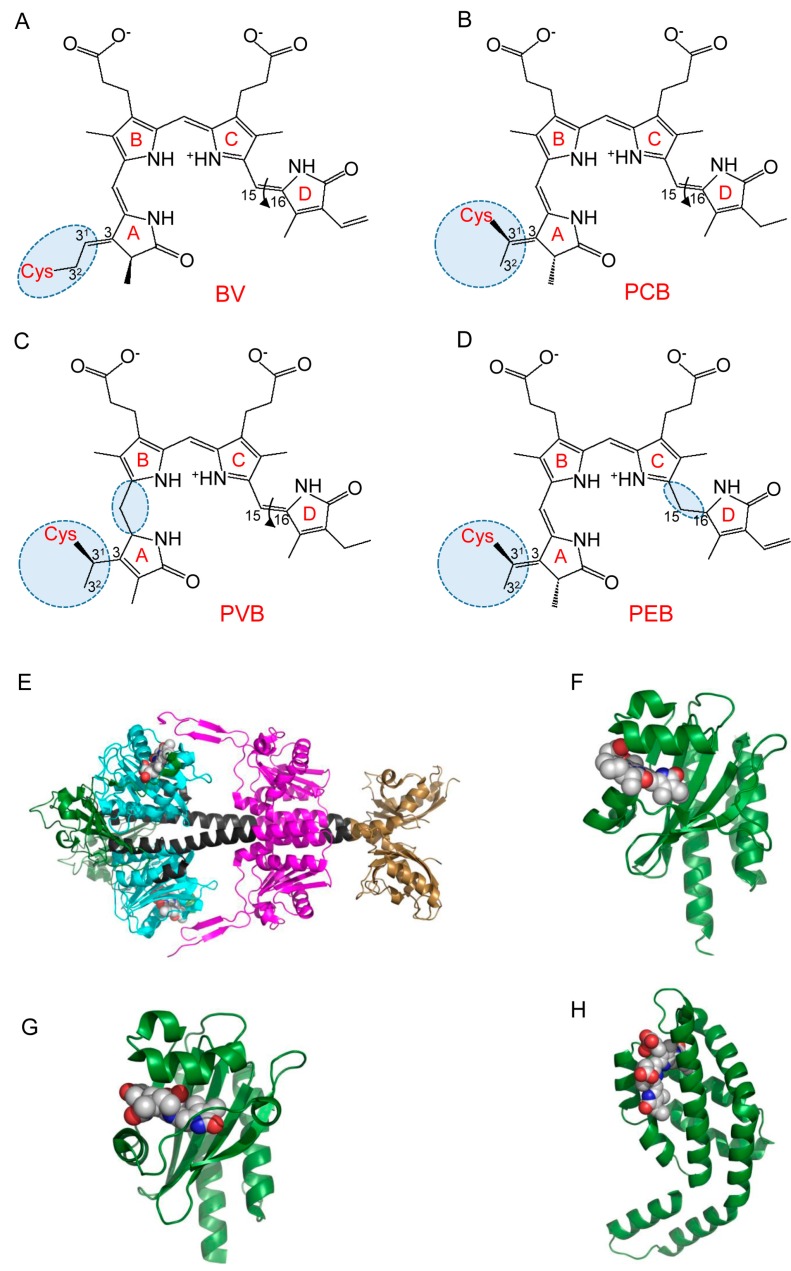
Chromophores and structures of selected bacterial photoreceptors. Linear tetrapyrrole chromophores are: (**A**) biliverdin (BV); (**B**) phycocyanobilin (PCB); (**C**) phycoviolobilin (PVB); and (**D**) phycoerythrobilin (PEB); (**E**) Crystal structure of BphP XccBphP from *Xanthomonas campestris.* PAS domain is shown in green, GAF in cyan, PHY in magenta and HisK in brown (PDB ID: 5AKP); (**F**) Crystal structure of CBCR AnPixJg2 from *Anabaena* sp. PCC 7120 in red-light-absorbing state (PDB ID: 3W2Z); (**G**) Crystal structure of CBCR TePixJg2 from *Thermosynechococcus elongatus* in green-light-absorbing state (PDB ID: 3VV4); (**H**) Crystal structure of APC B from *Synechocystis* PCC 6803 (PDB ID: 4PO5). BV and PCB chromophores in panels E–H are shown as spheres.

While BphPs incorporate BV plant phytochromes whereas Cphs (cyanobacterial phytochromes) can incorporate phytochromobilin (PΦB) and phycocyanobilin (PCB). Upon binding, a linear tetrapyrrole forms a thioether covalent bond with a Cys residue located in either the PAS (in BphPs) or the GAF domain (plant phytochromes) (Figure 1A–D). PCB and PΦB are found only in cyanobacteria and plants, in contrast BV is present in mammalian tissues due to its constant production from heme by heme oxygenase (HO). In mammals, BV level varies between tissues and substantially increases during oxidative stress [26]. 

Linear tetrapyrroles consist of four pyrrole rings that form a conjugated π electron system. Upon light absorption, the chromophores undergo Z/E isomerization around C15=C16 planar methine bond located between pyrrole rings C and D. The flip of the ring D around the methine bond provokes slight rotation of the entire chromophore relative to the protein cavity. Consequently, pyrrole rings B, C and D interact with different sets of amino acid residues in Pr and Pfr states [24]. These conformational changes are further propagated throughout the PCM and cause the rotation and activation of the downstream output domains [21].

Most likely, an incorporation of the chromophore into phytochrome proceeds through two successive steps: the non-covalent binding to GAF domain and the formation of thioether bond between a side chain of ring A and a conserved Cys residue located in either PAS or GAF domain. In bacterial phytochromes (Figure 1E), which are the most red-shifted among phytochromes, BV binds to the Cys residue via a C3^2^ carbon atom, whereas, in the plant and cyanobacterial phytochromes, PΦB and PCB bind to a C3^1^ carbon atom of the side chain of ring A [27]. Therefore, the absorption spectra of these phytochromes in the Pr state (λ_max_ = 650 nm) are blue-shifted compared to that of BphPs (λ_max_ = 700 nm). Being the most red-shifted photoreceptors, BphPs possess maximum absorption at 750 nm in Pfr state. Most BphPs adopt biologically inactive Pr state in darkness, whereas a subset of so-called bathy BphPs exists in the ground inactive Pfr state. Interestingly, bathy BphPs initially bind to BV in Pr form, which then converts into Pfr form [28]. 

BphPs weakly fluoresce upon light absorption in their Pr form [29,30], whereas Pfr form is non-fluorescent due to its very short excited-state lifetime, resulting in negligible quantum yield [31]. Deletions of N-terminal extension of PAS domain and PHY domain can increase BphP1 fluorescence by impairing Pr→Pfr photoconversion. All BphPs photoconvert between far-red and NIR parts of spectra, whereas CBCRs undergo very diverse photocycles, covering all visible spectra.

The modular structure and spectral properties of BphPs allowed their use as templates to engineer NIR FPs. Truncation and mutagenesis of DrBphP resulted in the first NIR FP, termed IFP1.4 [32]. This protein was applied to whole-body imaging, however, required the supply of exogenous BV. The latter can affect redox homeostasis and perturb intracellular environment [33]. The second NIR FP, termed iRFP713, was generated from RpBphP2 and was engineered to very efficiently incorporate endogenous BV. That is why iRFP713 proved to be superior for numerous in vivo applications [34]. Next, four spectrally distinct proteins that cover a large part of NIR spectrum, termed iRFP670, iRFP687, iRFP702, iRFP720, were developed by mutagenesis of RpBphP6 and iRFP713. Further improvement of BphP-derived NIR FPs was achieved by engineering a series of the monomeric miRFPs from RpBphP1 template [35] and mIFP from *Bradyrhizobium* sp. BrBphP [36].

### 2.2. Structure and Properties of Cyanobacteriochromes

CBCRs are a group of phytochrome photoreceptors found in cyanobacteria only [37]. Unlike other phytochromes, CBCRs require only a GAF domain for attachment of a tetrapyrrole chromophore [38]. It is considered that CBCRs initially bind PCB as a chromophore, but some dual-Cys CBCRs can then isomerize PCB into phycoviolobilin chromophore [18,39,40]. In heterologous expression systems, some CBCRs can also autocatalytically incorporate phytochromobilin and phycoerythrobilin [40,41,42,43]. Furthermore, several CBCRs can covalently incorporate as chromophore not only PCB but also BV [42,44,45,46]. 

**Figure 2:**
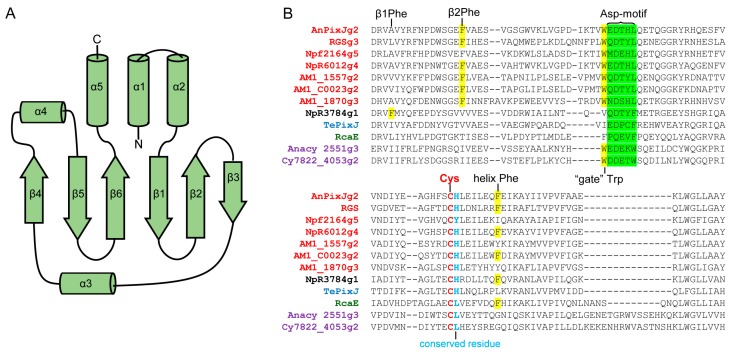
Structural properties of cyanobacteriochromes (CBCRs): (**A**) topology diagram of CBCR GAF domain; and (**B**) sequence alignment of chromophore-binding pockets in CBCRs. Representative CBCRs of the red/green subfamily (red), NpR3784 subfamily (black), DXCF subfamily (blue), green/red subfamily (green), and far-red/orange subfamily (purple) are included. Key CBCR residues are highlighted: Asp-motif is in green; β1 Phe, β2 Phe, helix Phe and “gate” Trp are in yellow; chromophore-binding Cys is in red; and conserved residue following chromophore-binding Cys is in blue.

Typically, CBCRs are mutlidomain photoreceptors, however, for simplicity, it was proposed referring to CBCR as an isolated photosensory domain [47]. CBCR domain adopts the typical GAF domain fold, structurally related to knotted PAS-GAF-PHY and unknotted GAF-PHY phytochromes. The tetrapyrrole-binding pocket of CBCR GAF domain is formed by anti-parallel β-sheets, β1–β6, together with α-helices, α3, α3′ and α4. The N-terminal helices α1, α2 and *C*-terminal helix α5 extend to other domains [48,49] (Figure 1F,G and Figure 2A). Commonly, α4 helix contains a “canonical” or “first” Cys residue that is connected to the C3^1^ atom of ring A via a thioether bond. Dual-Cys CBCRs, in addition to this canonical Cys, have a second conserved Cys residue that during photoconversion can reversibly bind to the chromophore [40,48,49,50]. Comparing BphPs, in the CBCRs the pyrrole ring A is more exposed to solvent and the unstructured loop that forms a knot is absent. Interestingly, on available CBCR crystal structures do not contain pyrrole water, which in BphPs participates in a hydrogen-bonding network [48,51].

In phytochromes and CBCRs, photoexcitation triggers a *Z*/*E* isomerization of the C15=C16 double-bond between pyrrole rings C and D. However, unlike phytochromes, CBCRs exhibit significant spectral diversity with a wide variety of photocycles, such as UV/blue [40], violet/green [52], violet/yellow [53], green/teal [54], teal/yellow [55], red/blue [56], green/red [57] and far-red/red [58]. To generate this spectral diversity, CBCRs use additional spectral tuning mechanisms including photochromism [57], PCB/phycoviolobilin (PVB) isomerization [59,60] and trapped-twist isomerization [61]. To date, a number of CBCRs subfamilies that utilize specific spectral tuning mechanisms and can be distinguished from each other by characteristic amino acid motifs and photocycle types have been identified.

Members of the green/red subfamily, RcaE and CcaS, are implicated in the regulation of complementary chromatic acclimation. These CBCRs in response to green and red light optimize expression of cyanobacterial light-harvesting phycobiliproteins [62,63]. Green/red CBCRs employ a photochromic photocycle, which combines tetrapyrrole chromophore photoisomerization with a subsequent shift in the chromophore’s p*K*a value. In the green-light-absorbing 15*Z* dark state the chromophore is deprotonated and in the 15*E* red-light-absorbing state it is protonated. Three conserved amino acid residues, called photochromic triad, facilitate the chromophore protonation state [64,65,66]. CcaS and its cognate response regulator CcaR were used to develop a light-regulated cell-recovery system [67] and several transcriptional regulatory optogenetic tools [68,69].

Dual-Cys CBCRs of DXCF subfamily, in addition to canonical Cys, contain a second Cys residue as a part of the DXCF amino acid motif. The DXCF motif includes an Asp residue found in other CBCRs, as a part of the conserved Asp-motif [70]. During photoconversion, the second Cys forms an additional thioether linkage with the C10 chromophore atom that blue-shifts the absorption [49,50,54]. Moreover, GAF domains of DXCF CBCRs usually are able to convert the PCB chromophore into PVB, which absorbs at shorter wavelengths [59]. TePixJ and Tlr0924, the typical members of this group, exhibit blue/green photocycle [48,49,50,71], whereas some other members of this subfamily have photoproducts absorbing teal, green or orange light [41,47,54].

Another subfamily of the dual-Cys group, termed insert-Cys CBCRs, contain second Cys residue as a part of CXXR/K amino acid motif. Typically, members of this CBCR subfamily exhibit violet/orange and UV/blue photocycles and do not isomerize PCB to PVB. Insert-Cys CBCRs are not closely related to DXCF CBCRs, and most likely this subfamily arose independently [40]. Insert-Cys CBCRs have an Asp-motif related to the Asp-motif of red/green CBCRs, and their second Cys is located in the insertion loop [70]. The members of this subfamily utilize dual-Cys photocycle. In the 15*Z* dark state, the second Cys forms a thioether linkage with the C10 atom of the chromophore. During photoconversion, this second covalent bond undergoes reversible breakage, resulting in 15*E* species with absorbance at longer wavelengths [40,70,72,73]. However, some members of this subfamily, such as NpF2164g2 and NpR1597g2, retain the second thioether linkage in both the 15*Z* dark and the 15*E* photoproduct state and exhibit UV/blue photocycle [40]. The DXCF CBCR, UirS, and its cognate response regulator UirR were used to develop a transcriptional regulatory tool activated by UV-violet light [74].

Members of DXCIP CBCRs group do not have the canonical first Cys residue, which usually forms a thioether linkage to C3^1^ atom of the PCB. For chromophore attachment, they utilize the second Cys residue only. This Cys is a part of the conserved DXCIP amino acid motif, which corresponds to DXCF motif of CBCRs from DXCF subfamily. These CBCRs exhibit 15*Z* green-light-absorbing dark state and slightly blue-shifted photoproduct [42].

A new type of dual-Cys CBCR, AM1_1186g2, which exhibits reversible photoconversion between a red-light-absorbing dark state and a blue-light-absorbing photoproduct, was found in cyanobacterium *Acaryochloris marina* (*A. marina*) In addition to canonical Cys, AM1_1186g2 contains a unique second Cys residue, which reversibly binds C10 chromophore atom in the blue-light-absorbing state. The wavelength separation between AM1_1186g2 photostates is the largest found to date [56]. 

Canonical red/green CBCRs have a red-light-absorbing 15*Z* dark state and green-light-absorbing photoproduct [75,76]. Members of this subfamily contain DX(Y/H)LQ amino acid motif and conserved Phe residues in β2-sheet and α4 helix [61]. The mechanism of red/green photocycle is not fully understood. According to a twist-trapped model, conserved Phe residue in β2 plays a crucial role in the formation of the blue-shifted photoproduct. After primary photoisomerization, steric interactions with Phe residue can constrain chromophore movement, trapping chromophore ring D in a twisted orientation. This model also explains a mechanism of photoconversion in teal-DXCF CBCR [61]. Another model, known as hydration model, proposes that photoisomerization leads to the rupture of the interactions between the chromophore and conserved Trp residue. The Trp acts as a gate allowing the influx of water molecules into the chromophore pocket. The resulting increased solvent accessibility, which may reduce the π-electron delocalization, leads to a blue-shift of the photoproduct [77]. However, it was shown that the chromophore protonation does not affect the properties of green-light-absorbing photoproduct of AnPixJg2 [78]. By ^13^C MAS NMR spectroscopy, it was also demonstrated that, during photoconversion, the electron density at chromophore rings C and D is increased. Consequently, it destabilizes the LUMO of AnPixJg2 in the Pg state, resulting in the blue-shifting of photoproduct [79]. Study of several red/green CBCRs from *Nostoc punctiforme* cyanobacteria revealed significant diversity in their excited-state lifetimes, photochemical quantum yields and primary photoproduct stabilities [80].

The CBCRs of NpR3784 subfamily exhibits red-light-absorbing 15*Z* dark state and photoproducts with variable absorption. Members of this subfamily contain the DXXF amino acid motif that corresponds to the DX(Y/H)LQ motif in canonical red/green CBCRs. To explain photocycle of the NpR3784 group, a twist-trapped model was proposed. However, this subfamily utilizes the unique set of Phe residues, different from the Phe residues of canonical red/green CBCRs [81].

Recently, new far-red/orange and far-red/red CBCR lineages were discovered [58]. Members of these subfamilies exhibit unique for CBCRs photocycles with 15*Z* far-red-light-absorbing dark state and 15*E* orange or red-light-absorbing photoproducts. Both subfamilies utilize PCB as a chromophore but their absorption peak in the dark state is at 725–750 nm. Some of the members of this subfamily exhibit weak far-red and NIR fluorescence [58].

Overall, CBCRs are attractive molecular templates for engineering NIR FPs (Table 1). Their photosensory module consists of only a single GAF domain. Individual GAF domains exhibit fluorescence when are heterologously expressed in *Escherichia coli* [46,58,82,83,84]. Furthermore, CBCRs AM1_C0023g2 and AM1_1557g2 were expressed in mammalian HeLa cells and exhibited NIR fluorescence upon cell treatment with PCB [46]. Contrary, BphP-based FPs consists of PAS-GAF [10,32,34,35,36,85] or even PAS-GAF-PHY domains [86]. CBCRs are likely monomeric; at least three CBCRs studied by NMR spectroscopy were monomeric in solution [50,70,87]. On the other hand, CBCRs utilize PCB as a chromophore, which is absent in animal cells. However, recently, three CBCRs from *A.marina* that covalently attach both PCB and BV were reported [44,45,46]. Two CBCRs with fluorescence at 730 and 740 nm were also described [44,58]. It is likely that these NIR CBCRs could be engineered into NIR FPs that will be more red-shifted than iRFP720, which is currently the most red-shifted NIR FP [10].

### 2.3. Allophycocyanins as Source of FPs

Allophycocyanins (APCs) are components of phycobilisomes, large light harvesting antenna complexes found in cyanobacteria and red algae [90]. Allophycocyanin α- and β-subunits (ApcA and ApcB), form heterodimers, that further assemble to disk-shaped (αβ)_3_ trimers. ApcA/ApcB trimers located in phycobilisome central cavity. More complex structures in phycobilisome core also contain ApcD, ApcF and linker protein ApcE (termed L_CM_). Due to their intrinsically fluorescent properties, allophycocyanin trimers widely used as labels in immunofluorescent techniques [91,92,93]. APCs utilize a PCB chromophore and form a thioether bond between the conserved Cys residue and C3^1^ atom of pyrrole ring A. ApcE attaches PCB autocatalytically through a thioether bond at the Cys196. In contrast, ApcA, -B, -D, and -F require so called bilin lyases for correct PCB attachment and form a thioether bond with conserved Cys81 residues [89,94]. However, the latter APCs can also bind chromophore without lyases with low efficiency [95,96]. The structures of all APCs are similar: an APC-like domain contains seven α-helices adopting globin-like fold, with N-terminal extension, which is mainly involved in oligomerization (Figure 1H) [89,97,98,99,100].

With co-expression of enzymes for PCB synthesis and appropriate bilin lyases, APCs are produced in *E.coli* as red FPs [94]. Remarkably, spectral properties of APCs could vary depending on their origin. For example, recombinant ApcD from *Synechocystis* sp. exhibits fluorescence with an emission peak at 642 nm, but ApcD from *Nostoc* sp. has fluorescence peak at 663 nm [89]. ApcE is a large membrane-associated protein with N-terminal APC-like domain. Its APC-like domain can be expressed in *E.coli* as FP with fluorescence maximum at 672 nm and quantum yield of 15% [83]. By truncating of the hydrophobic loop and N-terminal residues, there was engineered ApcE-based monomeric soluble FP, with emission at 663 nm and quantum yield of 6% [88]. ApcA from *Trichodesmium erythraeum* was engineered into far-red FP, smURFP. By directed mutagenesis, there were found amino acid substitutions that made TeApcA capable of binding BV without lyases. Although smURFP fluoresces in mammalian cells treated with an excess of exogenous BV, it is still quite dim compared to BphP-derived NIR FPs, such as miRFPs [7,101].

Recently, additional red-shifted APCs were found in NIR photoacclimated cyanobacteria. For example, ApcE2 from the *Synechococcus* sp., instead of the conserved Cys, has at the chromophore-binding site a Val residue. It results in non-covalent binding of the PCB chromophore and in the extremely red-shifted absorbance. A truncated monomeric ApcE2 (24–245) protein that exhibits fluorescence at 714 nm was engineered [102]. Similarly, ApcF2 with conserved Cys replaced by Tyr was found in *Chroococcidiopsis thermalis*. ApcF2 non-covalently binds PCB, absorbs at 675 nm and fluoresces at 700 nm [96]. 

APCs were optimized through natural evolution for efficient fluorescence resonance energy transfer and are brightly fluorescent. Overall, APCs are promising molecular templates to engineer more red-shifted NIR FPs (Table 1).

## 3. Engineering Approaches to Develop Near-Infrared Fluorescent Proteins

To date, BphP-based NIR FPs of different phenotypes were engineered by combining of rational design and directed molecular evolution (Table 2). The strategies used to engineer BphP-based NIR FPs could be applied for engineering NIR FPs from other bacterial photoreceptors. 

CBCRs structurally and functionally related to BphPs. Fluorescence and signaling compete in both CBCRs and BphPs. As light-sensing signaling molecules, they were evolved to maximize their quantum yield for entry into the photocycle. Hence, usually BphPs and CBCRs have relatively high extinction coefficient, but relatively low fluorescence quantum yield [6,103]. Suppression of chromophore photoisomerization leads to significant increase of BphPs fluorescence quantum yield and convert them to the bright fluorescence proteins [29]. APCs were evolved to collect and transfer light energy to the photosystems I and II, and hence they have relatively high extinction coefficient and fluorescence quantum yield. The main restriction for the APC-based NIR FPs construction is their pure expression, complicated chromophorilation and general tendency to oligomerization. 

The utility of biliprotein-based FPs in live cell and in vivo imaging depends on their intrinsic fluorescence intensity, protein expression level, folding and stability, as well as, specificity to BV chromophore [6]. Other important parameters including cytotoxicity, maturation time, photostability, pH sensitivity and oligomeric state also determined FP quality [104].

Availability of tetrapyrrole chromophore is essential for billiprotein-based NIR FPs. PCB, the natural chromophore of CBCRs and APCs, is found only in plant and cyanobacterial cells. However, in process of heme degradation, mammalian cells synthesize BV and it is abundantly present in animal tissues [26,105]. BV has the longest conjugated π-electron system among known linear tetrapyrroles and provides more NIR-shifted fluorescence. For engineering of fluorescent proteins from CBCRs and APCs, it is necessary to make them capable of incorporating BV. The combination of mutations that make CBCR capable of binding BV is not known. However, it was shown that Leu in position 337 is important for BV-binding property of AM1_1557g2 [44] and amino acid substitution Ser334/Gly increased the BV-binding activity of AM1_C0023g2 [46]. CBCRs and ApcE bind chromophore autocatalytically; the rest of APCs should be converted into lyases-independent form. Recently reported FP smURFP was evolved from APCα of *T. erythraeum* to autocatalytically attach BV. Amino acid substitution Asn42/Ile was necessary to make *Te*APCα capable of binding chromophore autocatalytically and three more mutations (Gly45/Ser, Arg61/His, Gln129/Lys) made it capable of binding BV [101]. 

### 3.1. Examples of Rational Design of Near-Infrared Fluorescent Proteins

Engineering BphP-based NIR FPs involves suppression of their photoconversion by deleting the PHY domain and introduce key mutations that stabilize Pr state [2,3,29,34,107]. Similar to BphPs, blocking of photoconversion leads to increasing of CBCRs fluorescence. Photochemically inert red/green CBCR Npf2164g5, found in *Nostoc punctiforme*, was unable to photoconvert; instead, it exhibited red fluorescence with a quantum yield of 10–15% [75]. Incorporation of PEB chromophore, which carries saturated bridge between pyrrole rings C and D and cannot isomerize [108], resulted in five-fold higher quantum yield of CBCR, Slr1393g3, as compared to its PCB chromophorilated form [43,84]. Suppression of CBCR photoisomerization can be achieved by mutation of the key amino acid residues, including those within the Asp-motif, conserved residue following chromophore-binding Cys, “gate” Trp residue and conserved Phe (Figure 2B). On the other hand, engineering of photoactivatable NIR FPs requires retaining of photoconversion. Two photoactivatable NIR FPs, termed PAiRFPs, constructed from bathy BphP, AtBphP2, contain its PAS, GAF and PHY domains [86]. A single GAF domain of CBCRs is sufficient for photoconversion and should allow engineering of smaller monomeric photoactivatable NIR FP.

BphP1-FP, iRFP670 and iRFP682 proteins, in addition to conserved Cys20 in the PAS domain, contain Cys residue in the conserved SPXH amino acid motif of the GAF domain (Cys253). NIR FPs with this additional Cys are blue-shifted, exhibit higher quantum yield and longer fluorescence lifetimes [27,109,110]. It was shown that mutants of such double-Cys NIR FPs, containing a single Cys either in the PAS or in the GAF domains, demonstrate different spectral properties. The variants with Cys in PAS domain had 35–40-nm red-shifted emission as compared to mutants carrying Cys in GAF domain. These findings made possible the rational design of brighter and spectrally distinct NIR FPs and were successfully applied to engineer spectrally distinct monomeric miRFPs [35]. In BphP-derived NIR FPs, the covalent binding of BV to Cys residue in the GAF domain via C3^1^ and C3^2^ chromophore atom leads to restricting ring A motion, resulting in a substantial increase of quantum yield [109]. Interestingly, these NIR FPs bind Cys in the GAF domain similarly to CBCRs, which also bind chromophore through C3^1^ carbon of ring A. By removing knot structure there was also engineered single-GAF fluorescent protein, termed GAF-FP, which is capable of covalent binding of both PCB and BV chromophores [12,111].

Monomeric BphP-based miRFPs were constructed by either structure-based monomerization or by selection of naturally monomeric BphP templates. The first approach was used to design Wi-Phy protein from DrBphP of *Deinococcus radiodurans*. Leu311 and Leu314 residues that created zipper interactions were replaced with negatively charged Glu residues. Moreover, a Phe145 residue that can participate in van der Waals interaction was replaced with Ser residue [112]. In contrast, RpBphP1 was chosen as a template to engineer miRFP670, miRFP703, miRFP709 and BphP1-FP. In the crystal structure of RpBphP1, the PAS and GAF domains are not involved in the dimerizing interface [19]. The characterization of these engineering NIR FPs confirmed their monomeric state [35,113]. 

APCs are module components of multidomain complexes with close inter-subunit interactions. Therefore, they usually form dimeric or oligomeric structure. However, recently, there was generated soluble monomeric mutant of ApcE, which exhibited autocatalytic chromophore binding, red-shifted absorption and emission spectra, and high quantum yield. The full-length N-terminal APC-like domain of ApcE (residues 1–240) is poorly soluble. However, deletion of a hydrophobic loop (residues 80–150) and 19 N-terminal residues resulted in a soluble 22 kDa monomeric FP [88]. 

### 3.2. High-Throughput Screening Approaches of Near-Infrared Fluorescent Proteins

Decay from the excited-state BV chromophore can occur via three competing processes: NIR fluorescence, C15=C16 double bond photoisomerization and non-radiative decay [31,114]. The latter one limits the fluorescence quantum yield of NIR FPs. Studies of several NIR FPs suggested that their C15=C16 photoisomerization was completely inhibited, but quantum yield was still rather low [109,114,115]. Hence, to improve quantum yield of BphP-based NIR FPs it is necessary to minimize non-radiative decay [109,114].

Crystallographic analyses and homology amino acid alignment allow identification of mutations important for inhibition of photoconversion or disrupting of a dimeric interface. However, an effect of multiple amino acid substitutions, which increase fluorescence quantum yield, enhance the rigidity of the chromophore packing and minimize non-radiative decay, is difficult to predict [112,116]. Moreover, the individually introduced these substitutions do not affect FP properties [30]. 

The cellular brightness of NIR FPs highly depends on their BV binding properties [2]. Unlike CBCRs and APCs, BphPs utilize BV as the natural chromophore. However, reported BphP-based NIR FPs exhibit the different efficiency of BV binding. Low specificity to BV led to the formation of undesirable complexes with PPIX and decreased cellular brightness [7,29,106]. As a result, FPs with comparable intrinsic fluorescence intensity, but different BV-binding efficiency, exhibit the large difference in cellular brightness [85]. Direct addition of exogenous BV can improve the brightness of suboptimal NIR FPs. However, this is undesirable because exogenous BV can affect cellular metabolism [117,118,119,120,121]. Co-expression of NIR FPs in mammalian cells with HO does not help to increase their cellular brightness [7]. To engineer NIR FPs applicable for mammalian cell expression, their BV-binding properties should be improved. For example, BphP-based NIR FPs of the iRFP and miRFP series were engineered to specifically incorporate endogenous BV in mammalian cells and can be readily applied to live cell imaging [7,10,34,35,36]. 

**Figure 3:**
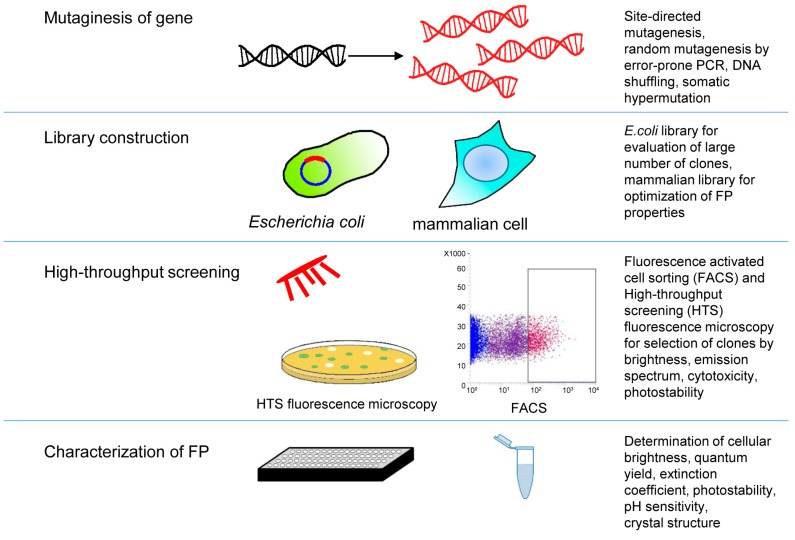
Molecular evolution steps, methods and techniques employed to develop near-infrared fluorescent proteins (NIR FPs). Left column lists typical molecular evolution steps, such as gene template mutagenesis, construction of library of mutants, methods of high-throughput screening, and characterization of advanced variants of NIR FP in vitro and in cells. Specific conditions, techniques and studied parameters of mutant for each molecular evolution step are indicated in the right column.

In initial steps of NIR FP engineering, rational structure-based approaches allow the development of mutant clones with improved properties. Further optimization of NIR FPs requires random mutagenesis followed by a selection of mutants with desired properties (Figure 3). A number of methods were proposed for mutagenesis of tagged sequence, but the most commonly used approach involves error-prone PCR techniques. Originally, error-prone PCR protocol was designed to reduce the fidelity of *Taq* DNA polymerase by increasing the concentration of MgCl_2_, addition of MnCl_2_ or nucleotides analogs and using unbalanced concentrations of nucleotides [122,123]. The main disadvantage of error-prone PCR that utilizes *Taq* DNA polymerase is the different frequency of base pair changes and bias towards transitions over transversions [124,125]. However, employing mutazyme II DNA polymerase specifically designed to construct error-prone PCR libraries allows generation of almost unbiased and diverse libraries [126]. Mutazyme II-based mutagenesis is widely used for engineering NIR FPs [27,35,85]. Moreover, to overcome limitations caused by biased polymerases, a sequence saturation mutagenesis method (SeSaM) was developed. SeSam allows broad distribution of nucleic acid mutations and can saturate any position of the target sequence [127]. SeSaM approach is effective but labor-intensive that hampers its use in FP engineering. DNA shuffling, a method for in vitro homologous recombination that generates diversity by combining useful mutations from individual genes [128,129] can also be used, particularly to engineer NIR FPs from CBCRs. Recombination between sequences of CBCRs from different subfamilies can be used to generate FP mutants with desired properties.

Not only quality but also the frequency of mutations is important. In libraries with low mutation rates, the majority of clones retain functional sequences but the number of unique mutations is low. High mutation rates result in many non-functional clones and increase probability of unique mutations. Only a few of clones from high-mutation-rates libraries are unique and functional. Identification of such mutants requires employing high-throughput screening approaches [130]. 

Basic high-throughput screening for NIR FP evolution is fluorescence-activated cell sorting. It enables easy screening of large libraries of up to 10^7^ clones. Molecular evolution of fluorescent proteins is mainly carried out via expression in *E. coli*, but yeast and mammalian cells also could serve as a host for evolving of FPs. Selections of clones from mammalian libraries could allow detection of bright, readily expressible and chromophrilated clones. However, using mammalian cells in directed evolution was hampered, because they have slow growth rates, low efficiency of stable gene integration, display variable expression levels and require time-consuming techniques. Employing small mammalian libraries can be an effective strategy to improve specific FP characteristics. For example, screening of photostable red FPs from a mammalian library generated by retroviral infection was reported [131]. 

*E. coli* remains the most widely used host for directed evolution. The major advantages of this common host are viability, rapid growth rates, high transformation efficiency and easy genetic manipulation. Production of bacterial photoreceptors in *E. coli* requires co-expression of HO for BV synthesis [34]. Co-expression of NIR FP and HO under control of different promoters allows regulation of BV level in host cells. Gradual reduction of BV level allows selecting FP mutants that bind BV with higher efficiency. 

## 4. Biological Applications of Near-Infrared Fluorescent Proteins

Modern imaging techniques have revolutionized basic biology because they allow non-invasive visualization of deep tissues. Imaging depth for particular tissue sample depends on its components that absorb and scatter light. The important parameter, called attenuation length, is the distance where the light intensity is decreased by 2.7-fold or, in other words, where about 63% of photons are absorbed by the tissue. This parameter depends on a particular sample, light wavelength and imaging modality, being about 100 µm or less for visible light. In contrast, attenuation length for NIR light is in the 1 mm range [132,133], therefore, the probability for a photon to travel unimpeded though the sample is significantly higher. Although GFP-like FPs are useful for single-cell studies, their use in macroscopic, high-throughput and animal studies is hampered by high autofluorescence and limited penetration depth of the visible light. Luciferase assays allow overcoming some of these difficulties but pose their own drawbacks. For example, in vivo luciferase assays depend on the level of cellular ATP and require injection of an exogenous substrate. Poor diffusion of the substrate in solid tumors and lack of ATP in dead cells limits data quantification and increases the variability of results.

### 4.1. Application of Near-Infrared Fluorescent Proteins to In Vivo Imaging

Initially, the performance of NIR FPs in deep tissue imaging was demonstrated in polyurethane mouse phantom that possesses light absorption and scattering properties similar to living animals [34]. In the phantom, iRFP713 was detected at the depth of up to 18 mm that provided higher signal-to-noise ratios than the best GFP-like red FPs. iRFP713 was also shown to be superior probe for photoacoustic imaging due to its large extinction coefficient (105,000 M^−1^·cm^−1^) and moderate quantum yield (~7%) [134]. Thus, in photoacoustic tomography, iRFP713 was detected at 9 mm depth in the model tissue. In mouse xenografts, iRFP713 was substantially brighter than EGFP and TagRFP657 that allowed detection and precise quantification of tumors on the depth of several millimeters with a lateral resolution of hundreds of micrometers. 

**Figure 4:**
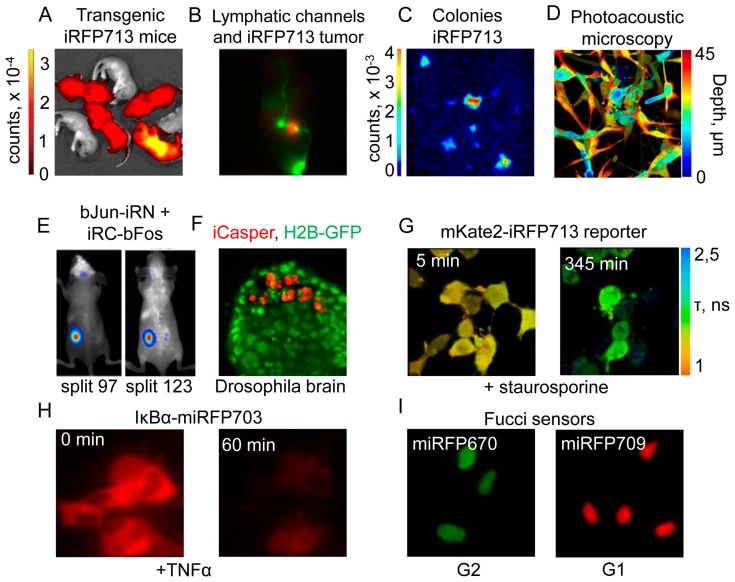
Applications of near-infrared fluorescent proteins. (**A**) Image of iRFP713 transgenic newborn mice and their wild-type non-fluorescent littermates (adapted from [26], copyright 2014 by the Japanese Association for Laboratory Animal Science); (**B**) Impaired lymphatic drainage due to metastatic spread. Lymphatic channels are shown in green and the iRFP713-labeled tumor is shown in red (adapted from [138], copyright 2014; available under the Creative Commons Attribution license); (**C**) Odyssey LI-COR scan of iRFP expressing 3T3 fibroblasts 16 days post transfection with cMyc/Ha-RasG12V. Fluorescent signal is shown in pseudocolor (adapted from [139], copyright 2014; available under the Creative Commons Attribution license); (**D**) Photoacoustic microscopy image of multiple layers of RpBphP1-expressing cells (right). Depth is shown in pseudocolor (adapted from [140]); (**E**) Detection of the bJun-bFos interaction in living mice expressing two split-iRFP713 constructs. Fluorescence pseudocolor images of tumor xenografts expressing bJun-iRN and iRC-bFos fusion proteins. Images for the constructs split at positions 97 (left) and 123 (right) of iRFP713 are shown (adapted from [141], copyright 2015 by Elsevier B.V.); (**F**) *Drosophila* brain cells co-expressing Histone-2B-GFP and the iCasper protease reporter. Apoptotic cells are shown in red (adapted from [142]); (**G**) Fluorescence lifetime images of cells expressing the mKate2-DEVD-iRFP713 reporter before (left) and after (right) induction of the apoptosis (adapted from [143], copyright 2016 by BioTechniques); (**H**) Stimulus-induced degradation of the NIR IκBα reporter, upon activation of the NF-κB pathway (adapted from [35]); (**I**) NIR Fucci cell cycle reporter in cells at different time points during cell cycle progression (adapted from [35]).

Transgenic mice expressing iRFP713 were healthy and expressed the protein in all tissues [26]. Their body mass, development and reproductive performance were unaltered. This correlated with previously observed low cytotoxicity of iRFP713 in transfected cells. NIR fluorescence was observed in all organs, being higher in lung, pancreas and liver that likely reflected the higher amount of HO in these tissues (Figure 4A).

NIR FP labeling is ideal for retinal cells because NIR light does not cause their photobleaching preserving their sensitivity and animal vision [135]. iRFP713 expression in hematopoietic cells is clinically relevant for the evaluation of cytotoxic effects of various compounds. Populations of erythrocytes, granulocytes and monocytes were undistinguishable in iRFP713-expressing and wild-type mice. Furthermore, simultaneous cell labeling with iRFP713 and GFP-like FPs opens new possibilities for multicolor imaging [136]. 

iRFP713 transgenic mice represent a powerful model for the transplantation experiments because NIR fluorescence from transplant can be detected in tissues. Transplantation of iRFP713 labeled pluripotent (iPS and STAP) cells allowed following cell fate after transplantation, their involvement in regeneration process, and the efficiency of medications [136]. 

Monitoring cell fate over the time often requires conditionally activated expression of probe in spatiotemporal manner. Recently, transgenic mice that bear iRFP713 under control of lox-stop-lox recombination element were established [137]. In these mice, iRFP713 fluorescence was detectable only upon Cre recombinase expression. It was demonstrated that iRFP713 is sensitive recombination marker for accurate assessment of Cre recombinase activity and recombination events in different tissues. Single iRFP713 allele integrated into the genome allowed monitoring recombination events by flow cytometry in single cells isolated from the animal tissues. With this technique, the amount of drugs inducing Cre activity administered to mice was significantly reduced.

### 4.2. Near-Infrared Fluorescent Proteins in Cancer Research

Non-invasive NIR fluorescent imaging of iRFP713-expressing cancer cells was used to monitor tumor progression in mouse models [144]. Lentivirus constructs encoding iRFP713 were used to generate labeled cell lines that were employed to monitor subcutaneous tumor growth in vivo. Moreover, intraosseous and intracranial implants were detected. The orthotopic xenograft tumor lung model was generated by labeling cancer cells with iRFP713 and Venus FPs [11]. In that case, NIR imaging allowed following cancer development in mice, whereas Venus fluorescence provided quantification of tumors ex vivo. This multicolor technique provided sensitivity to detect delicate changes of tumor development and evaluate the efficiency of therapeutic treatment that was challenging with conventional methods.

Metastatic spreading through lymphatic vessels and nodes in vivo was monitored by real time imaging of iRFP713-labeled cancer cells [138,145] (Figure 4B). In surgery, NIR fluorescent imaging also enabled accurate detection of cancer margin and image-guided resection of tumors. Multicolor labeling of tumors with iRFP713 and NIR-labeled antitumor antibodies helps to significantly reduce chances of residual disease and damage to critical structures proximal to tumor tissue [146]. 

Palette of five NIR FPs, such as iRFP670, iRFP682, iRFP702, iRFP713 and iRFP720, was used to label populations of cancer cells in vitro with confocal microscopy and flow cytometry and in vivo using whole-body imager [9,10]. A combination of bioluminescence and florescence increased the sensitivity of deep-tissue imaging. To achieve that, two chimeric luciferases consisting of *Renilla* luciferase fused with either iRFP670 or iRFP720 were engineered. Administration of the substrate caused a bioluminescence resonance energy transfer (BRET) from luciferase to iRFPs, resulting in NIR light bioluminescence [12]. 

### 4.3. Near-Infrared Imaging in High-Throughput Applications

Commercially available NIR scanners can detect NIR fluorescence in 700 and 800 nm channels allowing high-throughput quantification of cell number in vitro and ex vivo. The linear correlation between the iRFP713 signal and cell number allowed quantifying cells over several orders of magnitude that was impossible with other biomarkers [139]. Interestingly, iRFP713 signal did not increase in non-proliferating cells. The high speed of fluorescence analysis and absence of the requirement to collect and lyse cells make this method more robust than luciferase assay. Moreover, iRFP713 enables real-time monitoring of cell growth in various conditions, saving cost and time. In colony-based transformation assays, iRFP713 was employed for high-throughput screening of medicinal drugs. Because expression of a majority of NIR FPs of the iRFP family is non-toxic for cells, they are robust markers for evaluation of drug treatment on cell proliferation (Figure 4C). 

High-throughput NIR fluorescent assay is particularly useful in veterinary medicine to determine elevated levels of BV upon pathological states in avian and reptilian species [147]. Binding of BV to iRFP713 results in increase detected fluorescence with plate reader. This assay allows accurate determination of BV concentrations in the micromolar range. 

Some heavy metals can compete with BV chromophore for binding to NIR FP apoprotein, preventing the formation of covalent bond between the chromophore and Cys residue. Therefore, NIR FPs can determine the presence of heavy metals in various environmental studies. For example, IFP1.4 detects nanomolar concentrations of mercury in vitro. However, in cells, its sensitivity was substantially lower [148], perhaps due to neutralization of heavy metals by thiol-containing compounds, such as glutathione.

### 4.4. Near-Infrared Fluorescent Proteins in Microbiology and Parasitology

To date, a majority of the bacterial infection studies is based on the detection of bioluminescence in bacteria. Novel NIR fluorescence-based assays depend on the external source of light and could give higher background than luciferase, but they do not require administration of luciferase substrate. Therefore, iRFP713-expressing *Lactococcus lactis*, *Lactobacillus plantarum* and *E. coli* bacteria are promising tools for imaging of gastrointestinal microflora in vivo [149]. These bacteria were used to evaluate the time needed for food transit through the gastrointestinal tract, providing additional insights into the process of digestion. Moreover, the combination of iRFP713 and iRFP682-expressing bacteria enabled simultaneous detection of two different bacteria species in the animal using linear spectral unmixing. In future, labeled bacteria can be used as intra-intestinal factories for production and targeting of biologically active molecules. In parasitology, the introduction of iRFP713-expressing *Leishmania* parasites allowed their monitoring in mice and screening of medical drugs in vivo [150].

### 4.5. Near-Infrared Fluorescent Proteins in Advanced Imaging Techniques

Nevertheless, dimeric NIR FPs, such as iRFPs, possess limitations because of their oligomeric state that can interfere with normal function of a tagged protein. Therefore, tagging proteins with monomeric NIR FPs are preferable for imaging of intracellular structures, especially those possessed dynamic properties. For these purposes, tagging with miRFP670, miRFP703 and miRFP709 was used to visualize fine intracellular structures beyond the diffraction limit in super-resolution structured illumination microscopy [35]. Labeling of intracellular proteins with monomeric TagGFP2, mCherry and miRFP703 enabled multicolor super-resolution wide-field imaging. 

The introduction of NIR FPs accelerated the development of advanced imaging techniques. For example, fluorescence molecular tomography (FMT) and diffuse tomography (DT) allow 3D reconstruction of organs and tissues at the centimeter depth [151]. In living animals, organs and closely located tumors were resolved by DT using the combination of iRFP670 and iRFP713 [10]. Another method, called time-domain imaging technology (TD) possess increased sensitivity and higher signal-to-background ratio, compared to epifluorescence techniques. It measures fluorescence decay after delivery of short (10^−11^–10^−10^ s) laser pulses to sample, which allowed decreasing autofluorescence. Using this method, three tumors labeled with iRFP670, iRFP702 and iRFP720 were simultaneously detected [152]. Application of X-ray computed tomography and NIR imaging allow determining the exact position of a tumor inside the brain [152]. 

Photoacoustic tomography detects ultrasound waves generated by thermal expansions resulting from the thermal relaxations of periodically excited chromophores. Application of RpBphP1 to photoacoustic imaging allowed developing differential photoacoustic techniques, such as reversible-switchable photoacoustic tomography (RS-PAT) and reversible-switchable photoacoustic microscopy (RS-PAM) [140]. These techniques are based on the difference in extinction coefficients of RpBphP1 in Pfr and Pr states at 780 nm. The absorbance of BphP1 in Pfr state at 780 nm is five-fold higher than in Pr state, which generates significant differences in photoacoustic signal upon illumination of the sample with 780 nm light. RS-PAT detected metastasis in living mice at 1 cm depth and RS-PAM resolved multiple layers of RpBphP1-expressing cultured cells (Figure 4D). 

Photoactivatable and photoswitchable NIR FPs are a good alternative to constitutively fluorescent FPs in applications that require spatiotemporal labeling and tracking of proteins, organelles or cells. For that purposes, photoactivatable PAiRFP1 and PAiRFP2 are the optimal choices. PAiRFPs can be selectively and non-invasively photoactivated and visualized in vivo with a signal-to-background ratio higher than for constitutively fluorescent FPs. In the darkness, fluorescence of PAiRFPs exponentially decreases with time, allowing multiple photoactivation cycles [86].

### 4.6. Biosensors and Reporters Designed from Near-Infrared Fluorescent Proteins

NIR FPs are applied to engineer biosensors and reporters for detection of intracellular metabolites and protein-protein interactions (PPIs). Split protein reporters were created by splitting iRFP713 per two parts at the unstructured loop spanning PAS and GAF domains. The resultant constructs, termed iSplit, was used in bimolecular fluoresce complementation assay (BiFC) to detect PPI in HeLa cells and in vivo. Later, other split iRFP713 pairs were constructed for visualization of PPI between bJun and bFos proteins (Figure 4E) [141]. These pairs were employed also to monitor dynamics of PPI between viral and cellular proteins upon virus infection. Another split reporter, called IFP PCA was generated by splitting of IFP1.4. The split IFP1.4, termed IFP PCA, demonstrated apparent reversibility in yeast and mammalian cells but possessed significantly decreased brightness compared to iSplit. IFP PCA was useful to determine dissociation constants for PPIs in living cells.

Spectrally distinct miRFP670 and miRFP709 split pairs were generated from the respective monomeric NIR FPs [35]. These two mSplits have excellent spectral compatibility for simultaneous use in microscopy or flow cytometry. miSplits were used to detect mRNA distribution in live mammalian cells. Interestingly, both mSplits share the same PAS domain, but their GAF domains differ. Therefore, single PAS domain can interact either with mGAF670 or with mGAF709, reconstituting miRFP670 or miRFP709, respectively. Therefore, it is possible to discriminate between two different PPIs by using two mSplits. They also can be applied to monitor protein distribution between two compartments. 

Proteolysis is an important process that regulates cell fate, ontogenesis and pathogenesis. NIR FPs were used to engineer fluorescent and FRET-based protease reporters. FRET protease reporters, such as mKate2-DEVD-iRFP713 and eqFP650-DEVD-iRFP713, were exploited to detect apoptosis in mammalian cells, following activation of caspase-3 [143]. Another reporter, called iProtease, detects changes in fluorescence intensity upon proteolytic digestion of non-fluorescent precursor [142]. iProtease can be used in applications where utility of FRET-based reporters is limited by poor signal and necessity of image processing. iProtease was used for visualization of protease activity in cultured mammalian cells and in tumorigenesis in *Drosophila* embryos (Figure 4F,G).

Dynamic changes of intracellular protein levels upon posttranslational modification can also be monitored with NIR FPs. For example, activation of NF-κB signaling pathway was detected with IκBα-miRFP703 biosensor [35]. Upon activation of this pathway, the biosensor was targeted for degradation, resulting in fluorescence decrease in cultured cells treated with TNF-α. In mice, this occurred during the acute liver inflammation induced by injection of lipopolysaccharide (Figure 4H). 

NIR Fucci sensor (fluorescence ubiquitination-based cell cycle indicator) is used to visualize dynamics of cell cycle progression. This sensor was engineered by fusing spectrally distinct miRFP670 and miRFP709 to Cdt1 and Gem proteins, respectively. Accumulation of miRFP670-hGEM fusion was obtained during S/G2/M cell phases, whereas miRFP709-Cdt1 fusion was accumulated during G1 cell phase (Figure 4I). [35]. Later, smURFP and IFP2.0 pair was exploited in similar approach. However, for this pair, an addition of exogenous BV chromophore was required for optimal imaging [101]. 

## 5. Conclusions

Progress in the development of advanced light sources and imaging instrumentation has allowed non-invasive visualization of ongoing processes deeper in animal tissues. Moreover, design of NIR FPs has accelerated the development of advanced imaging techniques including diffuse optical tomography, photoacoustic tomography and bioluminescence imaging. NIR FPs possess excellent spectral compatibility with blue-light-activated optogenetic tools, allowing spatiotemporal control and visualization of intracellular processes. NIR FPs also complement GFP-like FPs, substantially extending a color palette available for high-throughput applications, live cell and in vivo imaging. Moreover, multicolor imaging allows simultaneous visualization of several biological processes. 

Recent achievements in the engineering of BphP-based NIR FPs together with their structural and functional studies provided a solid background for rational design and directed molecular evolution of novel NIR probes. The described above different families of bacterial photoreceptors share common chromophore binding moiety, incorporate the same chromophores and have rather similar photoconversion mechanisms. Therefore, novel NIR probes can be designed using the already tested for BphP-based NIR FPs approaches, such as inhibition of Pr↔Pfr photoconversion, reducing non-radiative decay and enhancing efficiency of chromophore incorporation.

CBCRs are attractive templates for NIR FP engineering due to their small size, monomeric nature and absence of knot structures. These properties make expression and genetic manipulations of CBCRs extremely robust. Notably, the most NIR-shifted bacterial photoreceptors are found among CBCRs. They can be employed to engineer probes extending the NIR region currently covered by BphP-based NIR FP. 

CBCRs can be also used to design small photoactivatable NIR FPs consisting of a single GAF domain. The difference in absorption and emission maxima between two photostates of CBCRs can be very large, exceeding 200 nm. This property can dramatically increase a signal-to-background ratio in deep-tissue differential imaging. 

APCs are also promising molecular templates because they are naturally brightly fluorescent. The main obstacles for engineering of NIR FPs from APCs are poor expression, requirements of lyase for chromophore incorporation and oligomerization. Likely, advanced engineering approaches should be developed to overcome these obstacles in future. 

The development of NIR FPs of small size and increased molecular brightness will advance biological studies at different scales, from single molecule to whole organisms. We anticipate that, in near future, NIR FPs, reporters and biosensors will be employed in high-throughput in vivo applications, in transgenic animal models and in various preclinical studies.

## Figures and Tables

**Table 1:** Spectral properties of molecular templates from bacterial photoreceptors.

Bacterial Photoreceptor	Host	Size (Amino Acid Residues)	Chromophore	Absorbance (nm)	Em (nm)	Quantum Yield, %	Reference
Anacy 2551g3	*Anabaena cylindrica*	195	PCB	716, 730	740	1.2	[58]
Cyan7822_4053g2	*Cyanothece* sp.	192	PCB	714, 732	736	<1.0	[58]
AM1_1557g2	*Acaryochloris marina*	165	PCB	649	676	1.7	[46]
AM1_1557g2	*Acaryochloris marina*	165	BV	700	730	0.3	[46]
AM1_C0023g2	*Acaryochloris marina*	151	PCB	650	879	3.0	[46]
AM1_C0023g2	*Acaryochloris marina*	151	BV	699	718	0.2	[46]
slr1393g3	*Synechocystis* sp.	162	PCB	650, 539	672, 616	6.0	[83]
Npf2164g5	*Nostoc punctiforme*	170	PCB	662, 640	n.a.	10.0	[75]
ApcE (36–240/Δ77–153)	*Nostoc* sp.	130	PCB	650	663	6.0	[88]
ApcD	*Nostoc* sp.	160	PCB	650	663	7.4	[89]

**Table 2:** Near-infrared fluorescent proteins and their reported applications in eukaryotic cells.

NIR FP	Ex, nm	Em, nm	EC, M^−1·^cm^−1^	QY, %	Molecular Brightness vs. iRFP713, %	Oligomeric State	Photostability in Mammalian Cells, *t*_1/2_, s ^1^	Brightness in HeLa Cells vs. iRFP713, % ^2^	Reference
IFP1.4	684	708	92,000	7.7	114	dimer	70	8.0	[32]
iRFP713 (aka iRFP)	690	713	98,000	6.3	100	dimer	960	100	[34]
PAiRFP1	659 ^3^	703 ^3^	67,100	4.8	64	dimer	n.d.	25	[86]
PAiRFP2	692 ^3^	719 ^3^	63,600	4.7	60	dimer		25	[86]
IFP2.0 ^4^	690	711	98,000	8.1	80	dimer	108	7.9	[85]
iRFP670	643	670	114,000	12.2	225	dimer	290	119	[10]
iRFP682	663	682	90,000	11.1	162	dimer	490	105	[10]
iRFP702	673	702	93,000	8.2	124	dimer	630	61	[10]
iRFP720	702	720	96,000	6.0	93	dimer	490	110	[10]
smURFP	642	670	180,000	18	551	dimer	300	1.0	[101]
BphP1-FP	640	669	60,000	13.0	126	monomer	n.d.	n.d.	[27]
GAF-FP	635	670	49,800	7.3	59	monomer	n.d.	2.0	[12]
mIFP	683	704	82,000	8.4	74	monomer	54	14	[36]
Wi-Phy2	696	719	118	8,7	n.d.	monomer	n.d.	n.d	[106]
miRFP670	642	670	87,400	14.0	198	monomer	155	72	[35]
miRFP73	674	703	90,900	8.6	127	monomer	394	37	[35]
miRFP79	683	709	78,400	5.4	69	monomer	192	30	[35]

The dimeric and monomeric NIR FPs are listed chronologically within each group. ^1^ The fluorescence decay curves obtained from transfected HeLa cells were normalized to absorbance spectra and extinction coefficients of FPs, spectrum of the lamp, and transmission of an excitation filter. ^2^ Determined as effective NIR fluorescence in HeLa cells without exogenous BV and after normalization to the fluorescence of co-transfected EGFP. ^3^ Corresponds to the photoactivated state. ^4^ Although IFP was originally reported to be a monomer [85], it was later shown to be dimer [6,36]. *Abbreviations:* FP, fluorescent proteins; NIR, near-infrared; EC, extinction coefficient; QY, quantum yield.

## Abbreviations

APC	Allophycocyanin
BphP	Bacterial phytochrome photoreceptor
BV	Biliverdin
CBCR	Cyanobacteriochrome
FP	Fluorescent protein
HO	Heme oxygenase
iRFP	Near-infraRed Fluorescent Protein
miRFP	Monomeric near-infraRed Fluorescent Protein
NIR	Near-infrared
PCB	Phycocyanobilin
PCM	Photosensory core module
PEB	Phycoerythrobilin
PPIX	Protoporphyrin IX
PΦB	Phytochromobilin
PVB	Phycoviolobilin
